# White button mushroom interrupts tissue AR-mediated TMPRSS2 expression and attenuates pro-inflammatory cytokines in C57BL/6 mice

**DOI:** 10.1038/s41538-021-00102-6

**Published:** 2021-08-02

**Authors:** Xiaoqiang Wang, Desiree Ha, Ryohei Yoshitake, Shiuan Chen

**Affiliations:** Department of Cancer Biology, Beckman Research Institute, City of Hope, 1500E Duarte, Duarte, 91010 CA USA

**Keywords:** Nuclear receptors, Chemokines

## Abstract

White button mushroom (WBM) is a common edible mushroom consumed in the United States and many European and Asia-Pacific countries. We previously reported that dietary WBM antagonized dihydrotestosterone (DHT)-induced androgen receptor (AR) activation and reduced myeloid-derived suppressor cells (MDSCs) in prostate cancer animal models and patients. Transmembrane protease serine 2 (TMPRSS2), an androgen-induced protease in prostate cancer, has been implicated in influenza and coronavirus entry into the host cell, triggering host immune response. The present study on C57BL/6 mice revealed that WBM is a unique functional food that (A) interrupts AR-mediated TMPRSS2 expression in prostate, lungs, small intestine, and kidneys through its AR antagonistic activity and (B) attenuates serum pro-inflammatory cytokines and reduces MDSC counts through its immunoregulatory activity. These findings provide a scientific basis for translational studies toward clinical applications of WBM in diseases related to TMPRSS2 expression and immune dysregulation.

## Introduction

White button mushroom (WBM) accounts for 90% of the total edible mushrooms consumed in the United States and many European and Asia-Pacific countries^1^. Our clinical phase I trial in prostate cancer patients indicated that dietary WBM powder suppressed prostate-specific antigen (PSA) and reduced the number of myeloid-derived suppressor cells (MDSCs) in blood circulation^2^. We recently showed that chemicals in WBM antagonized dihydrotestosterone (DHT)-induced androgen receptor (AR) activation and PSA expression in prostate cancer cells and animal models^3^. Other researchers have reported that dietary WBM enhances the innate immune response against bacterial and/or viral infections by enhancing natural killer (NK) cell activity^4^, promotes maturation of bone marrow-derived dendritic cells^5^, and reduces pro-inflammatory cytokine (interleukin (IL)-6, tumor necrosis factor (TNF)-α, interferon-γ) production^6^. β-Glucans, the most abundant carbohydrate found in yeast and edible mushrooms, including WBM, are a well-established immune modulator that stimulates the proliferation of lymphocytes while reducing inflammatory factors^7^. Yeast and mushroom-derived β-glucans have also been reported to suppress MDSCs^8^ in cancer models, consequently enhancing immunity against tumor development^9^. Taken altogether, these findings suggest that chemicals in WBM exert both anti-androgenic and immunomodulatory effects.

In prostate cancer, a key event is the oncogenic activation of transmembrane protease serine 2 (TMPRSS2), via AR signaling^10,11^. Recently, TMPRSS2 has been implicated in host cell entry to several viruses, including influenza virus and coronavirus (severe acute respiratory syndrome coronavirus (SARS-CoV), Middle East respiratory syndrome-CoV, and severe acute respiratory syndrome coronavirus 2 (SARS-CoV-2))^12–14^. Therefore, targeting TMPRSS2 could be a novel therapeutic approach for these viral infections^15,16^. With findings by us and others, we thus hypothesized that dietary WBM may interrupt DHT-induced AR-mediated TMPRSS2 expression throughout the body, including the prostate, the classical androgen-responsive organ, as well as other organs, such as the lungs, kidneys, and small intestine. DHT pellet-bearing C57BL/6 male mice were used to examine the effect of WBM on AR-mediated TMPRSS2 expression. We also hypothesized that dietary WBM could suppress inflammatory factors and reduce MDSCs via β-glucan. C57BL/6 male mice fed with WBM or β-glucan were applied to study WBM-induced immunomodulation.

Our proof-of-concept study in mice confirmed that dietary WBM interrupts AR-mediated TMPRSS2 expression throughout the body, including prostate, lungs, small intestine, and kidneys. We also found that oral intake of WBM decreases pro-inflammatory factors and reduces the MDSC counts in both blood and spleen. These results provide a scientific basis for translational studies toward clinical applications of WBM in diseases, including viral infections that are related to AR-mediated TMPRSS2 expression and immune dysregulation.

## Results

### WBM suppresses DHT-induced AR-medicated TMPRSS2 expression in multiple mice organs

We recently reported that dietary WBM antagonized DHT-induced AR activation and PSA expression in prostate cancer animal models and mouse prostate glands, without observable body weight loss, hepatotoxicity, and nephrotoxicity^3^. C57BL/6 mice were used to test our hypothesis that dietary WBM may interrupt DHT-induced AR-mediated TMPRSS2 expression throughout the body, including organs such as the prostate, lungs, small intestine, and kidneys.

**Fig. 1:**
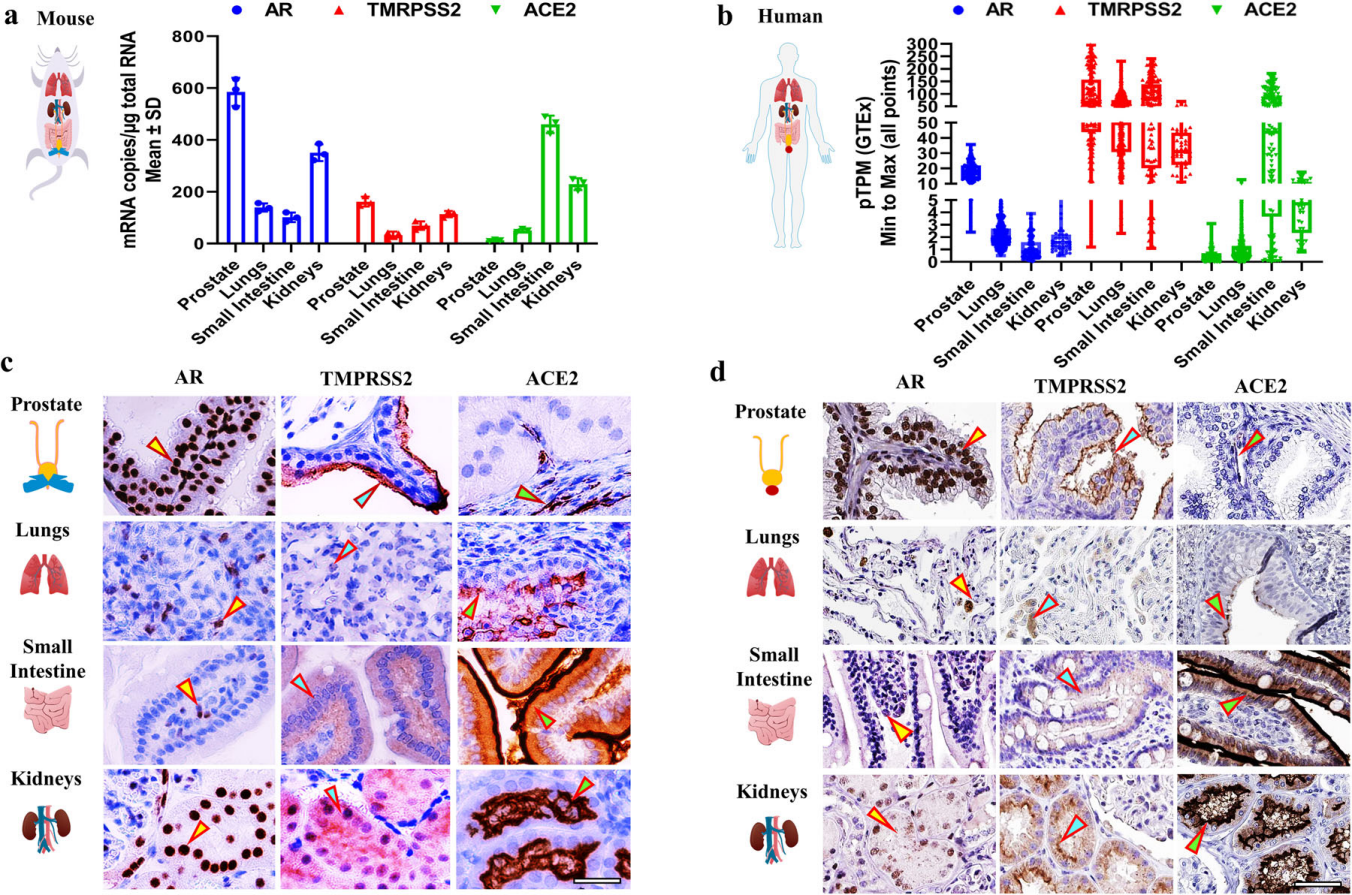
Tissue expression and localization of AR, TMPRSS2, and ACE2 in mice versus in humans. **a** The relative mRNA expression levels of *Ar*, *Tmprss2*, and *Ace2* in prostate (*n* = 3), lungs (*n* = 3), small intestine (*n* = 3), and kidneys (*n* = 3) in 8-week-old male C57BL/6 mice were quantified by qRT-PCR. qPCR values (mean ± SD) are expressed as mRNA copies/µg total RNA. **b** The mRNA expression levels of *AR*, *TMPRSS2*, and *ACE2* in the prostate (*n* = 152), lungs (*n* = 427), small intestine (*n* = 137), and kidneys (*n* = 45) in adult humans were extracted from the “The Human Protein Atlas/The Genotype-Tissue Expression (GTEx) Dataset”. GTEx dataset reports mRNA expression of each gene in human tissue as average pTPM. The data were presented by box plots showing all values. **c** The localization of AR, TMPRSS2, and ACE2 in the prostate, lungs, small intestine, and kidneys in 8-week-old male C57BL/6 mice were displayed by immunohistochemistry (IHC). **d** The IHC images of AR, TMPRSS2, and ACE2 in the prostate, lungs, small intestine, and kidneys in adult humans were extracted from the “The Human Protein Atlas/Protein Expression Summary” subcategory. In the database of The Human Protein Atlas, IHC staining was performed on normal human tissue. The scale bar of representative images is ×40 by 100 μm.

We first determined basal mRNA levels of *Ar*, *Tmprss2*, and *Ace2* in 8-week-old male C57BL/6 mouse tissues by quantitative reverse transcription polymerase chain reaction (qRT-PCR). *Ar* mRNA was highest in the prostate followed by the kidneys, *Tmprss2* mRNA was highest in the prostate, and *Ace2* mRNA was highest in the small intestine (Fig. 1a). We then determined the mRNA levels of the same three genes in adult human tissues by extracting data from “The Human Protein Atlas (HPA)/The Genotype-Tissue Expression (GTEx) Dataset”. Compared to their expression in mouse tissues, a similar expression pattern was observed in humans. Levels of mRNA for *AR* and *TMPRSS2* were highest in the prostate and *ACE2* mRNA was highest in the small intestine (Fig. 1b). To confirm and extend these results, we performed immunohistochemistry (IHC) to identify the specific cell types in the tissues that displayed expression levels for AR, TMPRSS2, and ACE2 in mice (Fig. 1c). To estimate the expression of these same proteins in human tissues, we extracted the IHC images from the staining reports in “HPA/Protein Expression Summary” database. The IHC images displayed the expression patterns and specific cell types that express AR, TMPRSS2, and ACE2 in normal human tissues (Fig. 1d). Similar to mRNA expression, AR, TMPRSS2, and ACE2 proteins were detected in all of the tested tissues at varying degrees. Mouse prostate glandular epithelial cells showed a high level of staining for AR (nucleus) and TMPRSS2 (apical lumen surface). A small proportion of basal cells that lie beneath the prostate epithelium exhibited a low level of ACE2 staining. Similar expression patterns of AR, TMPRSS2, and ACE2 were also observed in the human prostate (Fig. 1c, d—Prostate, indicated by arrows). In mouse lungs, strong staining for ACE2 was detected exclusively in the respiratory bronchiole epithelium, while moderate staining for AR and weak staining for TMPRSS2 was detected in alveolar cells, which is consistent with the expression pattern observed in the human lungs (Fig. 1c, d—Lungs, indicated by arrows). The mouse small intestine mucosa showed intense staining of ACE2 on the surface of the enterocytes, located on the top part of the villi. TMPRSS2 displayed a moderate staining in the crypt and the lower portion of the villi, except for the goblet cells, with the staining gradually diminishing toward the top of the villi. Meanwhile, AR staining was observed predominantly in lamina propria. These expression patterns mirror the results from human small intestinal tissue (Fig. 1c, d—Small Intestine, indicated by arrows). Concerning mouse and human kidneys, the AR staining was observed in the nuclei of tubular cells. TMPRSS2 was mainly expressed in the proximal tubular cells, while ACE2 was observed on the apical surface of the tubular cells (Fig. 1c, d—Kidneys, indicated by arrows).

**Fig. 2:**
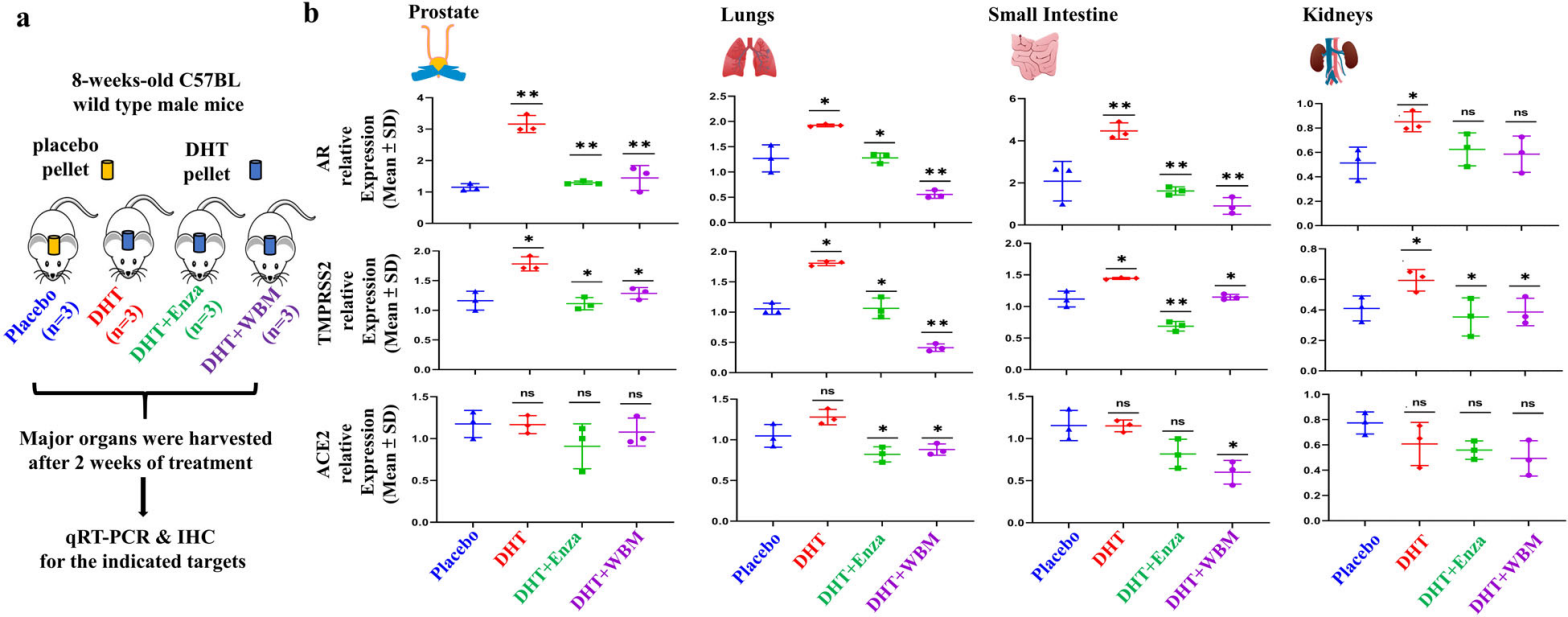
WBM suppresses DHT-induced AR-TMPRSS2 expression in multiple mouse organs. **a** Eight-week-old male C57BL/6 mice were subcutaneously grafted pellets (3 mice with placebo pellets versus 12 mice with DHT pellets). The 3 mice with placebo pellets were gavaged daily with 100 μL PBS for 2 weeks (Placebo group, *n* = 3). The 12 mice with DHT pellets were randomly divided into 3 groups and treated daily for 2 weeks as follows: 100 μL PBS (DHT group, *n* = 3), 300 μg enzalutamide (DHT + Enza group, *n* = 3), and 6 mg WBM (DHT + WBM group, *n* = 3). **b** The mRNA levels of *Ar*, *Tmprss2*, and *Ace2* in the prostate (*n* = 3), lungs (*n* = 3), small intestine (*n* = 3), and kidneys (*n* = 3) of each group were quantified by qRT-PCR. The target mRNA expression was quantified using the ΔΔCt method, as normalized to *Gapdh* transcript levels. The results were shown as mean ± SD. *p* Values were determined with one-way ANOVA (*p < 0.05, **p < 0.01. ns no significant difference).

Based on the above observations, we designed animal experiments (Fig. 2a) to validate in organs (prostate, lungs, small intestine, and kidneys) whether the expression of TMPRSS2 was upregulated in the presence of DHT and to determine whether WBM intake antagonized DHT-induced TMPRSS2 expression in these organs. Eight-week-old male mice implanted with placebo pellets were used as a baseline control (Ctrl) to compare to the mice bearing pellets containing DHT. Enzalutamide (Enza), a well-described AR antagonist, or WBM were given to DHT-treated mice as treatments. After 2 weeks of treatment, we harvested lungs, kidneys, small intestine, and prostate from each mouse for qRT-PCR and IHC analysis. Prostate was used as a positive Ctrl for systemic AR agonistic and antagonistic responses. As expected, *Ar* and *Tmprss2* expression was androgen-responsive in the prostate, as shown in DHT pellet-bearing mice. These expression levels were suppressed by treatment with either Enza or WBM (Fig. 2b—Prostate). Similar results on *Ar* and *Tmprss2* expression were obtained with the lungs, small intestine, and kidneys. The expression of *Ace2* did not display androgen responsiveness in the prostate and kidneys. However, *Ace2* expression displayed a trend of downregulation in the lungs and small intestine by Enza or WBM (Fig. 2b—Lungs, Small Intestine, and Kidneys).

**Fig. 3:**
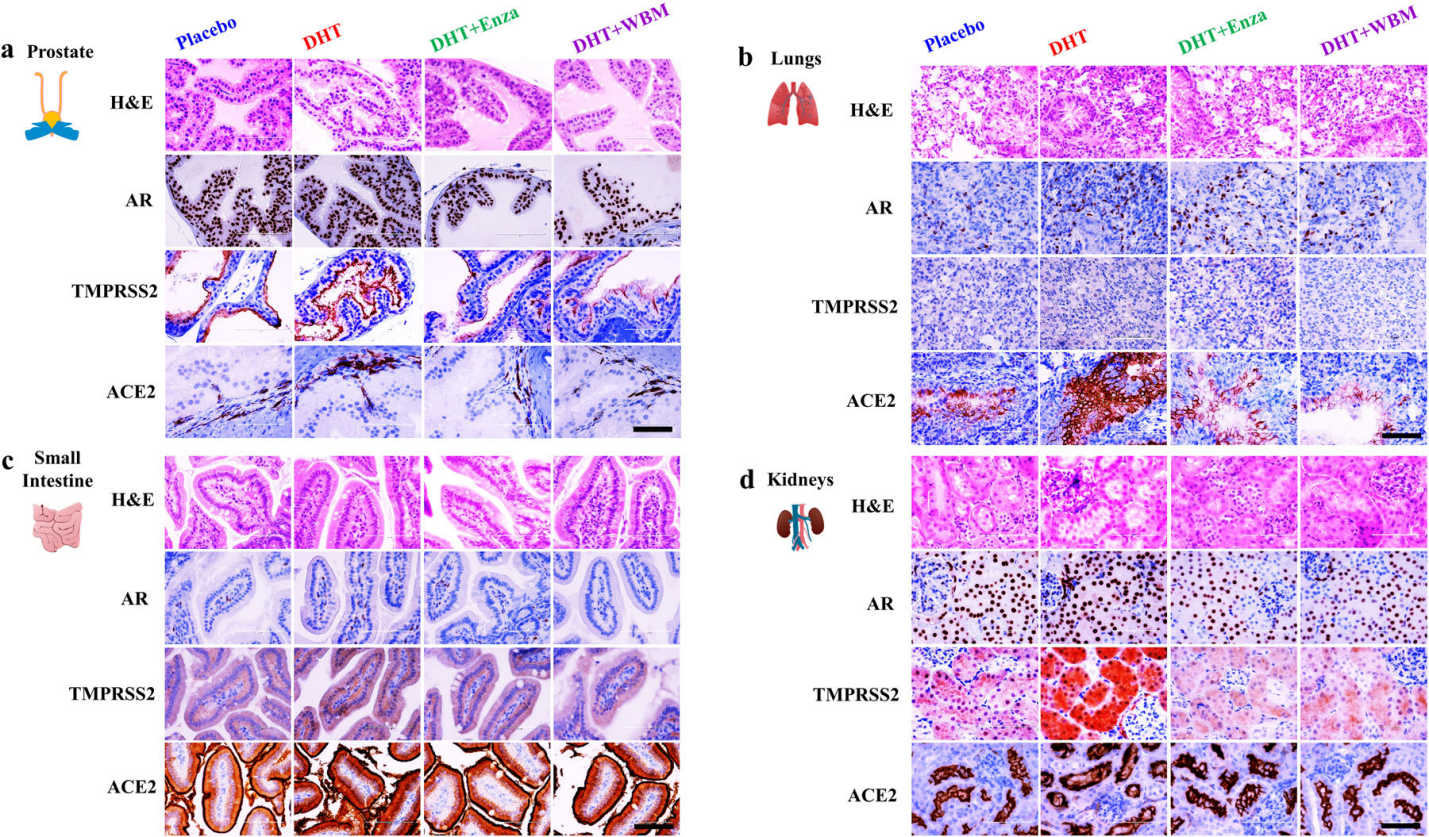
Histological evaluation of AR, TMPRSS2, and ACE2 expression in multiple mouse organs in response to DHT or WBM treatments. Eight-week-old male C57BL/6 mice were subcutaneously grafted pellets (3 mice with placebo pellets versus 12 mice with DHT pellets). The 3 mice with placebo pellets were gavaged daily with 100 μL PBS (Placebo group, *n* = 3). The 12 mice with DHT pellets were randomly divided into 3 groups and treated daily for 2 weeks as follows: 100 μL PBS (DHT group, *n* = 3), 300 μg enzalutamide (DHT + Enza group, *n* = 3), and 6 mg WBM (DHT + WBM group, *n* = 3). Hematoxylin and eosin (H&E) and IHC of AR, TMPRSS2, and ACE2 in the **a** prostate, **b** lungs, **c** small intestine, and **d** kidneys in each group. The scale bar of representative images is ×20 by 200 μm.

We also performed IHC to identify the specific cell types that displayed altered expression levels for AR, TMPRSS2, and ACE2 upon AR agonist or antagonist treatments in mice (Fig. 3). The hormone-responsive prostate glandular epithelial cells showed an increased number of AR-positive glandular epithelial cells (nucleus) and TMPRSS2 (apical lumen surface) proteins in response to DHT when comparing the placebo and DHT groups. This increase was suppressed by Enza or WBM, as seen when comparing the DHT group to the DHT + Enza/DHT + WBM groups. ACE2 protein, present in basal cells that lie beneath the prostate epithelium, displayed moderate changes from each treatment (Fig. 3a—Prostate). In the mouse lungs, the staining intensity of AR and TMPRSS2 in alveolar cells, especially ACE2 in the lung respiratory bronchiole epithelium, was increased upon DHT treatment, which was reduced by Enza or WBM treatment (Fig. 3b—Lungs). DHT induced the expression of AR, TMPRSS2, and ACE2 in the small intestine mucosa, but the addition of Enza or WBM was able to suppress these DHT-induced expression levels (Fig. 3c—Small Intestine). Concerning the mouse kidneys, DHT treatment increased the intensity of the AR staining, while Enza or WBM displayed a slight trend in attenuating DHT-induced AR expression. On the other hand, TMPRSS2 was induced significantly upon DHT exposure and was suppressed by Enza or WBM. We did not observe visible changes on ACE2 upon AR agonist or antagonist treatments (Fig. 3d—Kidneys).

Taken together, our analyses demonstrate that AR agonist or antagonist treatments influence the expression of AR and TMPRSS2, particularly in the lungs, small intestine, and kidneys. Slight changes to ACE2 by AR agonist or antagonist on the lungs and small intestine were also observed. Importantly, we experimentally demonstrated that WBM intake in mice disrupts DHT-induced AR-TMPRSS2 expression throughout the body, including organs such as the prostate, lungs, small intestine, and kidneys.

### WBM attenuates pro-inflammatory cytokines and reduced MDSCs in mice

**Fig. 4:**
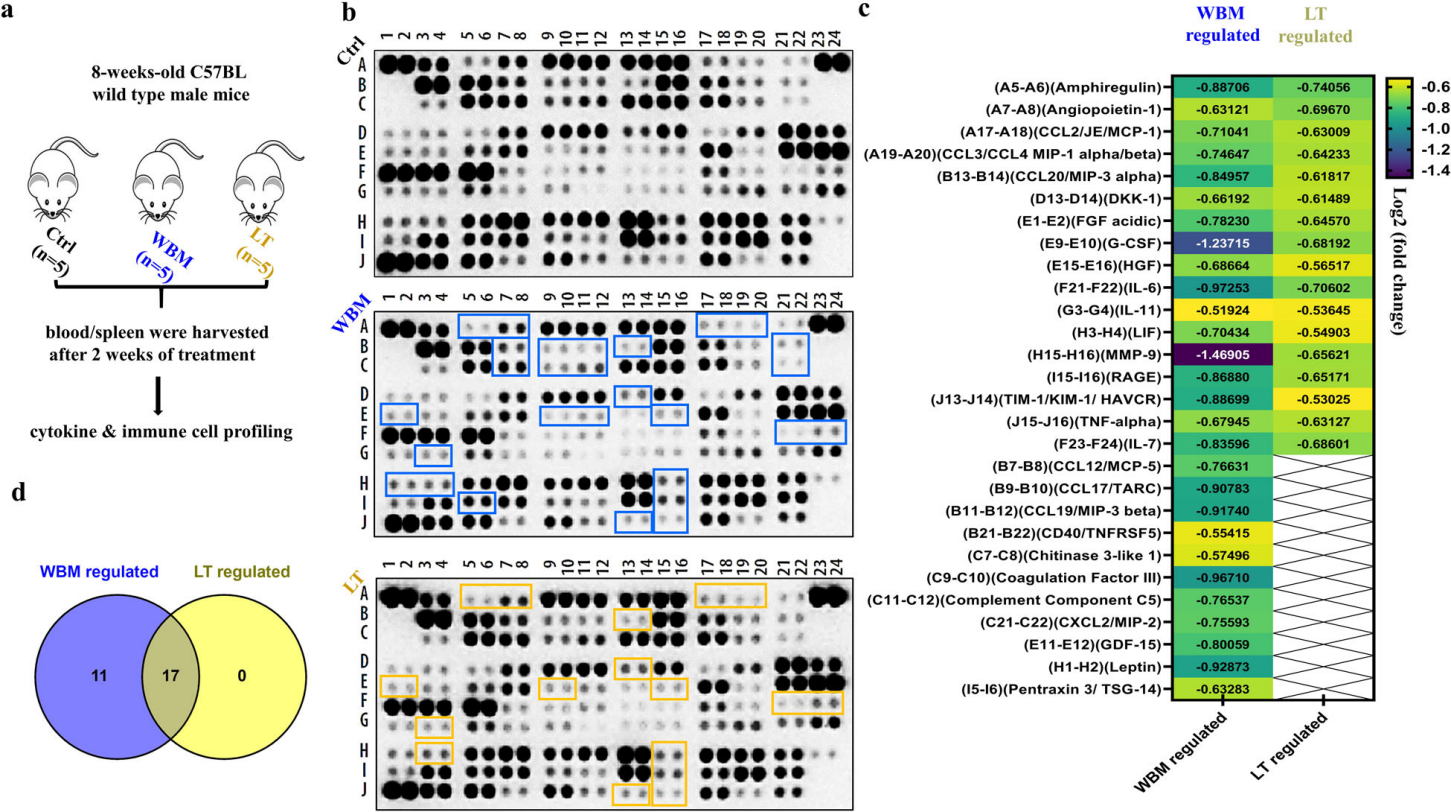
WBM attenuates pro-inflammatory cytokines in mouse serum. **a** Fifteen intact C57BL/6 mice were randomly divided into 3 groups and treated daily for 2 weeks as follows: mice in the Ctrl group (*n* = 5) were gavaged with 100 μL PBS, mice in the LT group (*n* = 5) were gavaged with 6 mg Lentinan, and mice in the WBM group (*n* = 5) were gavaged with 6 mg WBM. Serum was collected from each mouse. For the mice in the same treatment groups, their serums were pooled for a total of 500 μL (100 μL serum/mouse). Therefore, there was one pooled serum sample corresponding to each treatment group (Ctrl, WBM-treated, and LT-treated). **b** Proteome Profiler Mouse XL Cytokine Array Kit was used to semi-quantify 111 mouse cytokines, chemokine growth factors, and other soluble proteins in the pooled serum from each group. The image of dot spots represents the changing levels of cytokines. WBM-regulated cytokines were labeled with a purple-blue color, while LT-regulated cytokines were labeled with a light yellow color. **c** The Quick Spots software was applied to measure the mean spot pixel density, and significant changes (treatments versus control) were defined as upregulated (log2 fold change ≥0.5, *p* < 0.05) versus downregulated (log2 fold change ≤0.5, *p* < 0.05). **d** The overlapping cytokines between WBM versus LT treatment were displayed by Venn diagram using Venny 2.1.

β-Glucans are glucose polymers with a backbone of linear β-1,3-linked d-glucose molecules (β-1,3-d-glucan). They occur in the cell walls of plants and yeast and in fungi such as mushrooms^17^. β-Glucans derived from either yeast or mushrooms have been reported to enhance immunity against tumors or viruses^18^. Our clinical phase I trial in prostate cancer patients suggested that oral dietary WBM decreased circulating MDSCs, which are involved in immune response and inflammation^2^. We hypothesized that dietary WBM could suppress inflammatory factors and reduce MDSCs via β-glucan. In the present study, we designed an in vivo study (Fig. 4a) to investigate the immunoregulatory effects of WBM. Eight-week-old male mice were treated with WBM (which contains β-(1–3) glucan) or Lentinan (LT), a purified β-(1–3) glucan from a shiitake mushroom^19^. Untreated mice were used as Ctrls. After 2 weeks of treatment, we collected serum for cytokine profiling and isolated blood and spleens for MDSC characterization.

Mouse XL Cytokine Array detects 111 mouse cytokines, chemokine growth factors, and other soluble proteins. We identified a set of factors that were downregulated (log2 fold change ≤0.5, *p* < 0.05) by WBM or LT, as compared to untreated Ctrls, while upregulated factors (log2 fold change ≥0.5, *p* < 0.05) were not observed after both treatments (Fig. 4b, c). WBM suppressed the levels of 28 cytokines and LT suppressed 17 cytokines. All 17 cytokines downregulated by LT were also downregulated by WBM (Fig. 4d). The common 17 cytokines, suppressed by both WBM and LT (Fig. 4c, d), included molecules such as IL-6, IL-7, C-C chemokine motif ligand-2 (CCL-2)/monocyte chemoattractant protein 1 (MCP-1), CCL-3/CCL-4 macrophage inflammatory protein (MIP)-1α/β, TNF-α, and granulocyte colony-stimulating factor (G-CSF). Eleven cytokines were regulated by just WBM, and not by LT (Fig. 4c, d).

To further examine the identified cytokine profiles and the pathways underlying their synergism, we assessed the pathways by unbiased Gene Set Enrichment Analysis (GSEA). Twenty-eight WBM-regulated cytokines (including 17 common regulated factors between WBM and LT) were analyzed by the GSEA software and the WIKI pathways gene sets were selected. More importantly, Lung_Fibrosis and COVID19_Adverse_Outcome_Pathway were identified as the top two regulated pathways among the top five pathways (Table 1). As shown in Table 1, a cytokine trait that includes 11 factors (IL-6, TNF-α, G-CSF, MMP-9, HGF, FGF acidic, CCL-2/MCP-1, CCL-3/CCL-4 MIP-1α/β, C-X-C chemokine motif ligand-2 (CXCL-2)/MIP-2, and Pentraxin 3) was associated with the Lung Fibrosis pathway, while a subset of 6 cytokines (IL-6, TNF-α, G-CSF, CCL-2/MCP-1, CCL-3/CCL-4 MIP-1α/β, IL-7) was related to the coronavirus disease 2019 (COVID-19) Adverse Outcome Pathway.

**Table 1:** Top five pathways suggested by Gene Set Enrichment Analysis (GSEA).

Gene set name	*p* value	FDR *q* value	*k*/*K* value	Cytokine symbol
Lung Fibrosis	1.21e−24	7.11e−22	11/63	IL-6, TNF-α, CCL-2/MCP-1, CCL-3/MIP-1α, G-CSF, CXCL-2/MIP-2, MMP9, CCL-4/MIP-1β, Pentraxin 3, HGF, FGF acidic
COVID-19 Adverse Outcome Pathway	3.27e−16	9.59e−14	6/15	IL-6, TNF-α, CCL-2/MCP-1, CCL-3/MIP-1α, G-CSF, IL-7
IL-18 Signaling Pathway	2.68e−15	5.24e−13	10/279	IL-6, TNF-α, CCL-2/MCP-1, CCL-3/MIP-1α, CXCL-2/MIP-2, MMP9, CCL-4/MIP-1β, Pentraxin 3, CCL19/MIP-3β, CCL20/MIP-3α
Cytokines and Inflammatory Response	1.49e−14	2.19e−10	6/26	IL-6, TNF-α, G-CSF, CXCL-2/MIP-2, IL-7, IL-11
Toll-like Receptor Signaling Pathway	1.92e−10	1.87e−8	5/104	IL-6, TNF-α, CCL-3/MIP-1α, CCL-4/MIP-1β, CD40/TNFRSF5

Twenty-eight WBM-regulated cytokines (including 17 common regulated factors between WBM and Lentinan (LT)) were analyzed by GSEA and WIKI pathways gene sets were selected. Top 5 pathways are presented with *p* values (<0.05), False Discovery Rate (FDR) *q* values (<0.05), and *k*/*K* values [genes in overlap (*k*) to genes in gene set (*K*)].

**Fig. 5:**
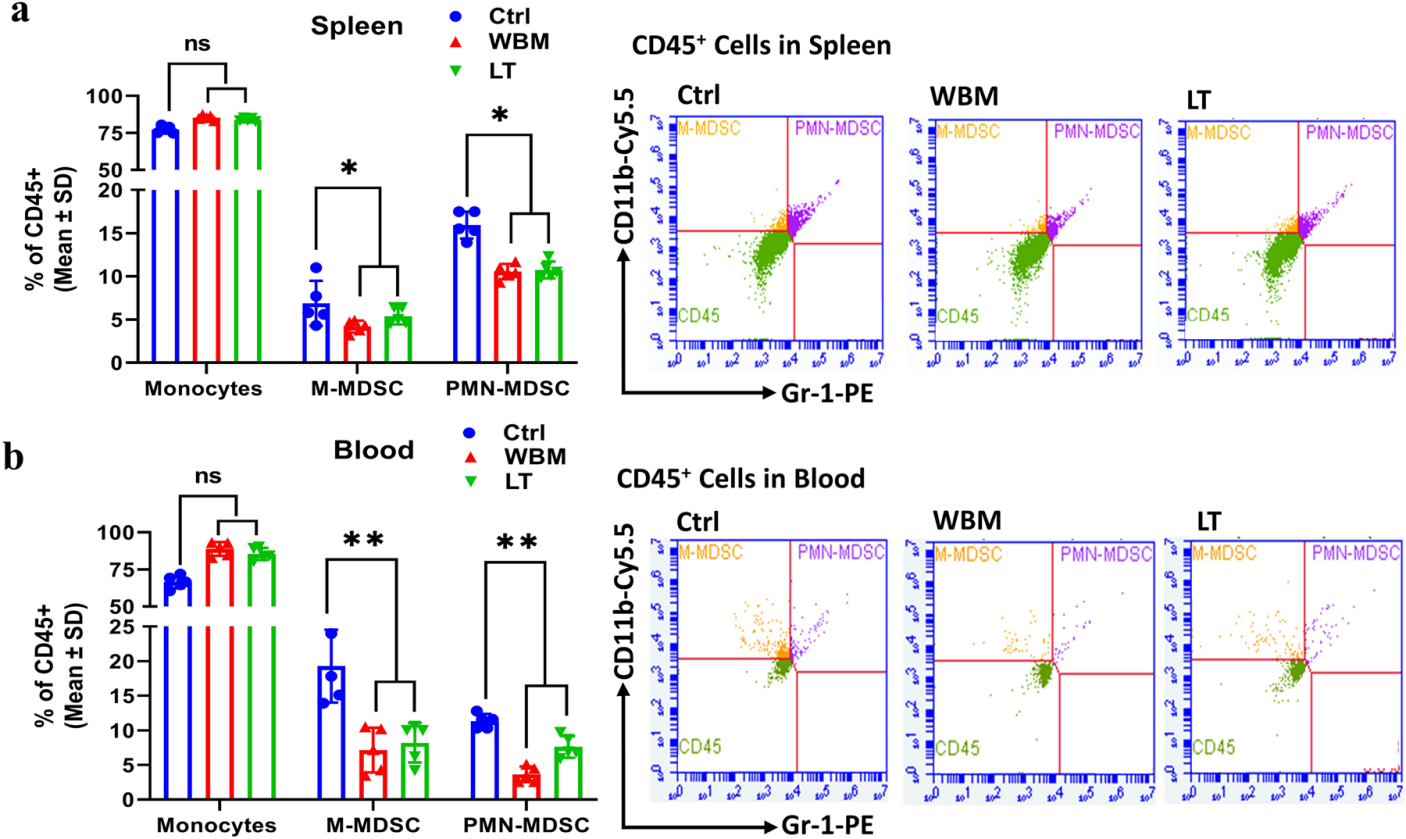
WBM decreases MSDC counts in mouse spleen and blood. Fifteen intact C57BL/6 mice were randomly divided into 3 groups and treated daily for 2 weeks as follows: mice in the Ctrl group (*n* = 5) were gavaged with 100 μL PBS, mice in the LT group (*n* = 5) were gavaged with 6 mg Lentinan, and mice in the WBM group (*n* = 5) were gavaged with 6 mg WBM. BD Accuri C6 Plus flow cytometer was used to identify myeloid-derived suppressor cells (MDSCs) in the **a** spleen and in **b** blood from mice in each group. BD Accuri C6 system software was used to analyze the data. Mouse monocytic MDSCs (M-MDSCs/CD45^+^/CD11b^+^/Gr-1^low/mid^) and granulocytic MDSCs (PMN-MDSCs/CD45^+^/CD11b^+^/Gr-1^high^) were gated to show the two population of MDSCs. The results are presented as mean ± SD. *p* Values were determined by one-way ANOVA (*p < 0.05, **p < 0.01. ns no significant difference).

To further characterize the immune responses to WBM treatment, we quantified the levels of MDSCs in the spleen (Fig. 5a) and blood (Fig. 5b) by performing multi-parametric fluorescence-activated cell sorting analysis. WBM or LT clearly decreased the total counts of monocytic (M)-MDSCs (CD45^+^/CD11b^+^/Gr-1^low/mid^) and polymorphonuclear (PMN)-MDSCs (CD45^+^/CD11b^+^/Gr-1^high^) in both peripheral blood and spleen (Fig. 5).

## Discussion

TMPRSS2 is an androgen-regulated serine protease involved in human prostate cancer progression and metastasis^10,11^. Molecular studies revealed that there are androgen-responsive elements within the promoter region of the TMPRSS2 gene, which likely accounts for its typical androgen-dependent transactivation. Therefore, the expression of TMPRSS2 in prostate cancer cells can be effectively suppressed by AR antagonist^11^. TMPSSR2 is also involved in facilitating the entry of several types of viruses, including influenza virus and SARS-CoV-2 into host cells^12–14^. Therefore, targeting TMPRSS2 could be a novel antiviral strategy to treat coronavirus and influenza virus infections^15,16^. More significantly, accumulating epidemiologic data indicate that the severity and progression of COVID-19 are greater in men than in women^20^. One hypothesis to account for this infection gender discrepancy is that viral entry is potentially enhanced in the lungs and other tissues of men through AR-mediated upregulation on TMPRSS2^21^. The inhibition of the androgen signaling axis impacts TMPRSS2 expression in the prostate gland, as well as throughout the body, including the lungs^22^. Therefore, repurposing anti-AR drugs that have been approved for prostate cancer, in the context of the COVID-19 pandemic, is one of the efforts that are currently being made^23^. Several clinical studies, including some employing large epidemiological cohorts, suggested that blocking androgen signaling might protect against COVID-19^24,25^. The clinical trial using AR antagonists, proxalutamide/GT0918, in COVID-19 is currently underway (ClinicalTrials.gov, NCT04446429. Anti-Androgen Treatment for COVID-19). The trial released the preliminary analysis of proxalutamide as a treatment for COVID-19 patients. The data showed that proxalutamide could significantly ameliorate symptoms and prevent hospitalization for COVID-19 patients^26^.

In the current study, we have shown that DHT regulates the expression of AR and TMPRSS2 in subsets of pulmonary and intestinal epithelial cells, as well as in kidney proximal tubular cells. The AR and TMPRSS2 levels are markedly elevated in the lungs, small intestine, and kidneys upon DHT exposure, while WBM and an AR antagonist, Enza, effectively repressed DHT-induced transcription of TMPRSS2 (Figs. 2 and 3). Regarding the androgen-induced TMPRSS2 regulation in lung epithelial cells, some previous data showed no effects of Enza on pulmonary TMPRSS2^27^. However, several studies have demonstrated that androgen regulates the expression of TMPRSS2 in subsets of lung epithelial cells, while treatment with Enza reduces TMPRSS2 levels in human lung cells^28^, thus inhibiting SARS-CoV-2 infections in vitro^29^. As compared to the numerous investigations of AR-mediated TMRPSS2 expression in the lungs, there are few reports of this regulation in other organs, such as the small intestine and kidneys^30^. One study described that, in castrated C57BL/6 male mice, TMPRSS2 staining was reduced in the bronchial epithelium of the lungs, columnar epithelium of the small intestine, and proximal convoluted tubules in the kidneys^31^. In our experiments, we observed that AR and TMPRSS2 levels were significantly elevated in the lungs, small intestine, and kidneys upon DHT exposure (Figs. 2 and 3). Our study using a male mouse model provided additional evidence that AR induced TMPRSS2 expression in the lungs, small intestine, and kidneys, while intake of WBM and Enza effectively repressed DHT-induced transcription of TMPRSS2 in those organs. By implications from the ongoing clinical trial with an AR antagonist for preventing COVID-19 infection, our studies suggest that WBM is a potential dietary intervention to prevent COVID-19 entry into the cells of the lungs, small intestine, and kidneys.

The severity and clinical outcomes of COVID-19 patients are due to not only the viral load but also to the host’s response that is triggered by viral entry and replication^32^. Severe COVID-19 infection is characterized as a pro-inflammatory status by high levels of inflammatory factors produced by hyperactive immune cells. The inflammatory factors include cytokines such as IL-6, TNF-α, and G-CSF and chemokines such as CCL2, CCL3/4, and CXCL-2. These, together with reactive oxygen species, have been recognized to induce acute respiratory disease syndrome, leading to lung fibrosis and possibly death^33,34^. Therefore, systematically alleviating this hyper-activated inflammatory state is crucial to improve the prognosis and outcome of COVID-19^35^. Several approaches such as IL-6 inhibitors and immune checkpoint inhibitors have been proposed to counteract the “cytokine storm” present in severe COVID-19 patients. However, the benefits, dose, and duration of these approaches remain to be validated^36^. Massive infiltration of mononuclear cells has also been detected in infected lungs, with parallel low levels of hyperactive T cells in circulation^34,37^. The immense migration of innate immune cells to the infected tissue, to control the viral replication, could contribute to the tissue damage and lead to multiple organ failures^38^. MDSCs constitute an important system for regulating these inflammatory and T cell responses^39,40^ and indeed MDSCs are increased in COVID-19 patients^41^. Increasing MDSCs during the early stages of COVID-19 were correlated with IL-1β, IL-6, IL-8, and TNF-α plasma levels, particularly in patients who required intensive care treatments, suggesting new therapeutic options geared toward MDSCs^42^.

Dietary β-glucans are suggested as an efficient, low-cost, and safe way to overcome the hyper-inflammatory status while balancing effective immune responses^43,44^. Indeed, β-glucans have been shown to exert antiviral properties and decrease the severity of both upper and lower respiratory tract viral infections in both animal and human studies^45,46^. β-glucans can reprogram innate immune cells results in immune enhancement by the activation of NK cells and CD4^+^ Th1 cells and suppression of the inflammatory response via downregulation of pro-inflammatory factors such as IL-6, CCL2, CXCL10, et al.^7^. Also, β-glucans have been shown to enhance anticancer immunity by suppressing MDSCs^8,9^. Our clinical phase I trial in prostate cancer patients also indicated that oral dietary WBM reduced the counts of MDSCs^2^. In the line of evidence, we hypothesized that dietary WBM has immunoregulatory effects by suppressing pro-inflammatory cytokines and MDSCs through β-glucans. To confirm our hypothesis, we demonstrated (Fig. 4) that WBM or LT, a shiitake mushroom-derived β-(1,3)-glucan, predominantly suppressed a panel of cytokines when compared to Ctrl. The cytokine signatures associated with treatments of WBM and LT greatly overlapped (15 out of 26 for WBM, 15 out of 15 for LT). More importantly, the unbiased GSEA analysis suggested that the shared cytokines included molecules that were associated with the Lung Fibrosis Pathway and the COVID-19 Adverse Outcome Pathway (Table 1). These cytokines, such as IL-6, IL-7, CCL-2/MCP-1, CCL-3/CCL-4 MIP-1α/β, TNF-α, and G-CSF, have been implicated in COVID-19 pathogenesis^47,48^. Besides, we also observed a subset of 11 factors that was selectively regulated by WBM. Among these, we pointed out a defined cytokine trait (CCL-12/MCP-5, CCL-19/MIP-3β, CXCL-2/MIP-2, etc.) of severe COVID-19 outcome^48^. As expected, when we characterized MDSCs in both blood and spleen from WBM- or LT-treated mice (Fig. 5), we observed the regulatory trend that both WBM and LT decreased the total counts of M-MDSCs and PMN-MDSCs in peripheral blood and spleen. Our results indicate that β-glucans are probably a major component of WBM that modulates immune function. In considering that WBM is a mixture of multiple components, we cannot rule out the possibility that additional chemicals besides β-glucans in WBM may potentially exert immunoregulatory activities.

**Fig. 6:**
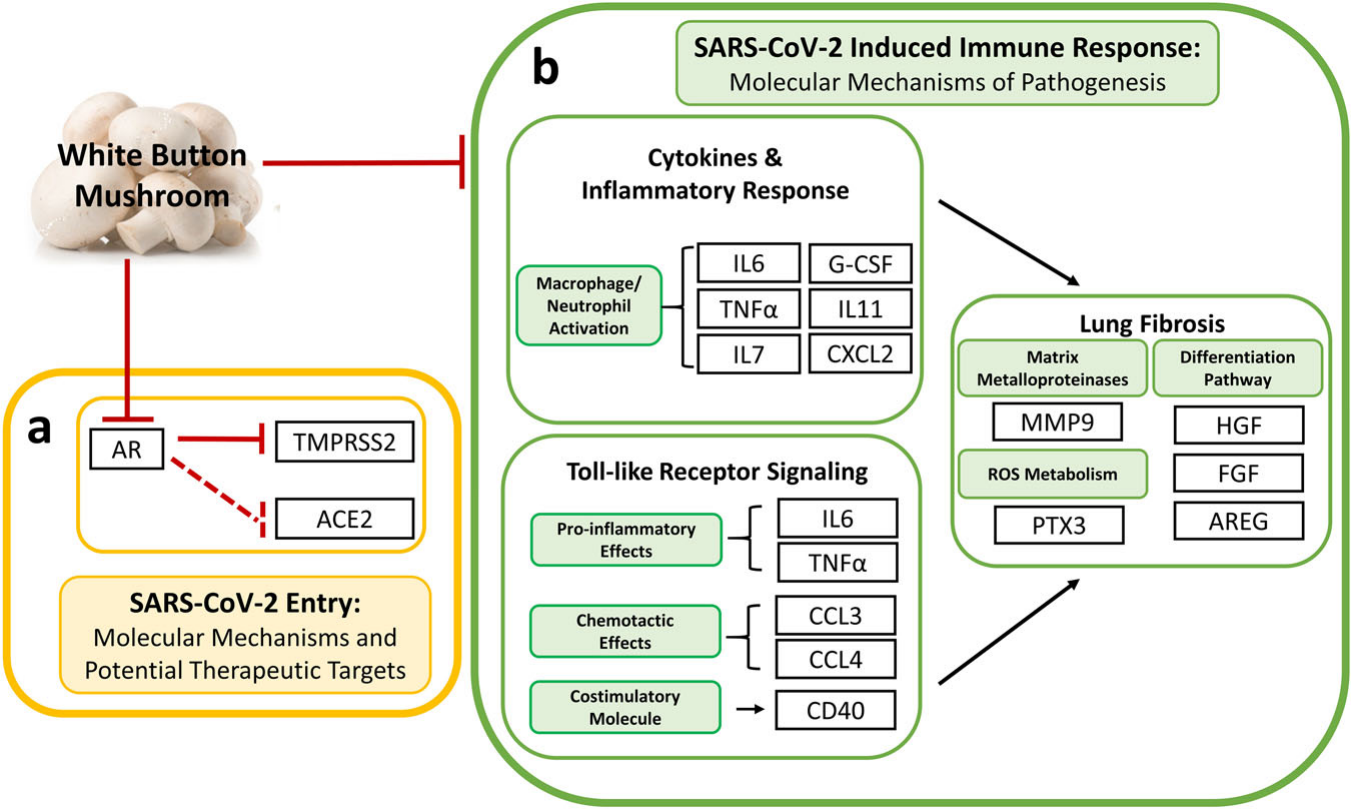
Hypothetical mechanisms of WBM as a dietary intervention for COVID-19. **a** WBM interrupts AR-mediated TMPRSS2 expression that is involved in viral entry, through its AR antagonistic activity, and **b** WBM attenuates serum pro-inflammatory cytokines and reduces MDSC counts that are involved in the host response to viral infection, through its immunoregulatory activity.

In conclusion, our preclinical studies on C57BL/6 mice have revealed that WBM is a unique food that (A) interrupts DHT-induced TMPRSS2 expression, through its AR antagonistic activity, and (B) attenuates serum pro-inflammatory cytokines and reduces MDSC counts through its immunoregulatory activity (Fig. 6). The data reported here suggest that WBM could be a useful dietary intervention in the COVID-19 pandemic. Our clinical trial using WBM with prostate cancer patients determined that a dose level of up to 14 g WBM powder/day (equal to 140 g fresh WBM) resulted in minimal side effects, mostly limited to abdominal bloating. Indeed, WBM intervention is considered safe with patient compliance at 98.6%. However, studies with animal models designed specifically for COVID-19 and trials on COVID-19-infected patients will be needed to confirm WBM’s efficacy.

## Methods

### Mushroom and chemical reagents

WBM extract was prepared via hot water extraction. Briefly, 6 g of freeze-dried WBM powder generated from 60 g fresh mushrooms were boiled in 1 L hot water for 3 h. The broth was centrifuged at 3000 × *g* for 30 min, twice, to collect the fraction of the supernatant. The liquid fraction was rotor-evaporated to dryness and then re-dissolved in 1 mL of hot water to produce a 6× mushroom extract (6 mg/µL)^3^. Enza, an AR antagonist, was purchased from Selleckchem (MDV3100, Selleckchem, Houston, TX). LT, a shiitake mushroom-derived immunomodulatory β-(1, 3)-glucan, was purchased from Carbosynth (FL33321, Compton, Berkshire, UK).

### Animal experiments

Eight-week-old male C57BL/6 mice (The Jackson Laboratory, Sacramento, CA) were used for the evaluation of WBM-mediated AR-TMPRSS2 suppression and immunomodulation. Mice were housed in an Association for Assessment and Accreditation of Laboratory Animal Care International-accredited Animal Resources Center. Animal research procedures were approved by the Institutional Animal Care and Use Committee at City of Hope (Approval Number: 15051) to ensure the ethical and sensitive care and use of animals in research and testing. All experiments performed in this study complied with the ethical regulations for animal testing and research.

To study WBM-mediated AR-TMPRSS2 suppression, in each experiment, 12 mice were subcutaneously grafted with DHT pellets (12.5 mg/60 days, Innovative Research of America, Sarasota, FL), while 3 mice were grafted with placebo pellets without DHT (Innovative Research of America, Sarasota, FL). The mice with placebo pellets were gavaged daily with 100 μL PBS with 1% carboxymethyl cellulose for 2 weeks. The 12 mice with DHT pellets were randomly divided into 3 groups and treated daily for 2 weeks as follows: 3 mice in Ctrl were gavaged with 100 μL PBS with 1% carboxymethyl cellulose, 3 mice in the Enza group were gavaged with 300 μg/mice Enza (at a dose of 10 mg/kg), and 3 mice in WBM group were gavaged at a dose of 200 mg/kg/day (average body weight of mice is 30 g, equal to 6 mg/mice/day) in 100 μL PBS with 1% carboxymethyl cellulose.

To study WBM-mediated immunomodulation, 15 mice were randomly divided into 3 groups and treated daily for 2 weeks as follows: 5 mice in the Ctrl group were gavaged with 100 μL PBS with 1% carboxymethyl cellulose, 5 mice in the LT group were gavaged with 6 mg/mouse LT at a dose of 200 mg/kg (average body weight of mice is 30 g, equal to 6 mg LT/mouse/day), and 5 mice in the WBM group were gavaged at a dose of 200 mg/kg (equal to 6 mg WBM powder/mouse/day in 100 μL PBS with 1% carboxymethyl cellulose.

Throughout the treatments, body weights were monitored every 2 days as an indicator of the mice’s overall health. At the end of the treatments, the mice were euthanized. The androgen-responsive organ (prostate) and other major organs (lungs, small intestine, and kidneys) were flash-frozen in liquid nitrogen and/or fixed with 4% paraformaldehyde. The expression levels of AR, TMPRSS2, and ACE2 were assessed by qRT-PCR and IHC. Whole-blood samples were collected and isolated into serum and cell pellets. Serum samples were stored at −80 °C for the cytokine array assay, while cell pellets were pre-fixed and stored at −80 °C for the flow cytometric assay. Spleens were collected to isolate splenocytes, which were then pre-fixed and stored at −80 °C for the flow cytometric assay.

### Quantitative real-time PCR

Total RNA was extracted using the RNeasy Mini Kit (QIAGEN, Germantown, MD) and was then used to synthesize cDNA with SuperScript IV VILO Master Mix (Thermo-Fisher, Grand Island, NY). qRT-PCR was performed in duplicates using SYBR Green Fast Master Mix (Quantabio, Beverly, MA) and analyzed on CFX manager 3.1 (Bio-Rad, Hercules, CA). The primers (Supplementary Table 1) used in the qRT-PCR were obtained from Primer Bank (https://pga.mgh.harvard.edu/primerbank) and synthesized by Integrated DNA Technologies. For the comparison of the target mRNA expression across various tissue types, we related copy numbers of each gene to total RNA concentration. qPCR values were expressed as mRNA copies/µg total RNA. For the comparison of the gene expression in the same tissue type, the target mRNA expression was quantified using the ΔΔCt method, as normalized to *Gapdh* transcript levels.

### IHC and histological analysis

Hematoxylin and eosin staining and IHC on formalin-fixed tissues were performed by the Pathology Core at City of Hope. Antibodies used in IHC included: AR (SP107, Sigma-Aldrich), ACE2 (ab108252, Abcam), and TMPRSS2 (ab92323, Abcam). Slides were first reviewed at ×10 magnification to identify areas of positive staining, followed by confirmation and quantification at ×20 magnification. Representative images were acquired using an Olympus BX46 microscope with a DP27 camera at magnifications of ×20 and ×40, with a scale bar of 200 and 100 μm, respectively.

### Expression levels and localization of AR, TMPRSS2, and ACE2 in human tissues

For the expression and localization of AR, TMPRSS2, and ACE2 in the human prostate, lungs, small intestine, and kidneys, the mRNA expression values and IHC images were obtained from “HPA” (http://www.proteinatlas.org/)^49^. The mRNA expression values were chosen from the “HPA/GTEx Dataset”. GTEx dataset reports mRNA expression of each gene in human tissue as average pTPM. Transcripts per kilobase million (TPM) is the normalization method for RNA-seq in GTEx dataset, TPM is for all transcripts in a sample that add up to 1 million mapped reads. pTPM is for all TPM values per sample scaled to a sum of 1 million TPM. There were 152 samples for prostate, 427 samples for lungs, 137 samples for the small intestine, and 45 samples for kidneys^50^. The IHC images were extracted from the “HPA/Protein Expression Summary” subcategory. The information on expression patterns and specific cell types that express respective genes were extracted from the staining reports in the “Protein Expression Summary” database. In this database, IHC staining was performed on normal human tissue. AR expression was detected with a rabbit antibody (1:250, HPA004733, Sigma-Aldrich) and validated with another rabbit antibody (1:500, GTX62599, GeneTex). ACE2 expression was primarily detected with a rabbit antibody (1:250, HPA000288, Sigma-Aldrich) and confirmed with a mouse antibody (1:5000, CAB026174, R&D Systems); TMPRSS2 expression was detected with a rabbit antibody (1:300, HPA035787, Sigma-Aldrich)^51^.

### Cytokine profiling assay and data analysis

Proteome Profiler Mouse XL Cytokine Array Kit (ARY028, Bio-Techne, Minneapolis, MN), a membrane-based antibody array, was applied to semi-quantify 111 mouse cytokines, chemokine growth factors, and other soluble proteins in serum. There were five mice in each treatment group (Ctrl, WBM-treated, LT-treated), and serum was collected from each mouse. For the mice in the same treatment groups, their serums were pooled for a total of 500 μL (100 μL serum/mouse). Therefore, there was one pooled sample corresponding to each treatment group (Ctrl, WBM-treated, and LT-treated). The array test was duplicated for each group. The assay was performed following the manual’s protocol. The scanned image of dot spots (Supplementary Fig. 1) was analyzed by the Quick Spots software (HLImage+ +, Western Vision Software, Salt Lake City, UT), which measured the mean spot pixel density. The significant changes (treatments versus Ctrl) were defined as upregulated (log2 fold change ≥0.5, *p* < 0.05) versus downregulated (log2 fold change ≤0.5, *p* < 0.05). The overlapping cytokines between the two treatments are displayed by Venn diagram using Venny 2.1. GSEA was used to identify pathways underlying cytokine synergism. The top five pathways determined by GSEA are presented with False Discovery Rate *q* values (<0.05), *p* values (<0.05), and *k*/*K* values [genes in overlap (*k*) to genes in gene set (*K*)].

### Flow cytometric assay

BD Accuri C6 Plus flow cytometer (BD, San Jose, CA) was used to identify MDSCs in the spleen and blood. BD Accuri C6 system software was used to analyze the data. Mouse MDSCs were identified by staining with the following panel of antibodies: fluorescein isothiocyanate-conjugated anti-mouse CD45 (30-F11, eBioscience, San Diego, CA), PerCP-Cy5.5-conjugated anti-mouse CD11b (M1/70, eBioscience, San Diego, CA), and phycoerythrin-conjugated anti-mouse Gr-1 Ly6G/Ly-6C (RB6-8C5, eBioscience, San Diego, CA). M-MDSCs (M-MDSCs/CD45^+^/CD11b^+^/Gr-1^low/mid^) and granulocytic MDSCs (PMN-MDSCs/CD45^+^/CD11b^+^/Gr-1^high^) were gated following the sorting strategies (Supplementary Fig. 2) to show the two distinct populations of MDSCs.

### Statistical methods and data analysis

Measurements were taken from distinct samples in each treatment. Results are shown as mean ± standard deviation (SD). All statistical analyses were performed using the GraphPad Prism software (version 9.1). The significance of the differences between the mean values was determined with one-way analysis of variance. *p* Values < 0.05 (*p < 0.05, **p < 0.01) were considered statistically significant and all tests were two-tailed.

### Reporting summary

Further information on research design is available in the Nature Research Reporting Summary linked to this article.

## Supplementary information

Supplementary Information

Reporting Summary

## Data Availability

The data supporting the findings reported herein are available on reasonable request from the corresponding author.
