# Supplementary material for: White button mushroom interrupts tissue AR-mediated TMPRSS2 expression and attenuates pro-inflammatory cytokines in C57BL/6 mice
Source: NPJ Sci Food. 2021 Aug 2;5:20. doi: 10.1038/s41538-021-00102-6 (PMC8329194; doi:10.1038/s41538-021-00102-6)

**Supplementary Information**

**Title**

White Button Mushroom Interrupts Tissue AR-mediated TMPRSS2 Expression and Attenuates Pro-inflammatory Cytokines in C57BL/6 Mice

**Authors**

Xiaoqiang WANG^1^, Desiree Ha^1^, Ryohei Yoshitake^1^, Shiuan Chen^1^**^*^**.

^1.^ Department of Cancer Biology, City of Hope, 1500 E. Duarte Rd., Duarte, CA 91010, USA

* Correspondence to: Shiuan Chen Ph.D.; Department of Cancer Biology, Beckman Research Institute, City of Hope, 1500 E. Duarte Rd., Duarte, CA 91010, USA. Phone: +1-626-256-4673; Email: [schen@coh.org](mailto:schen@coh.org)

**Supplementary Table 1.** All primers used in this study

| Name of Genes | Sequences (Forward) | Sequences (Reverse) |
| --- | --- | --- |
| Ar | 5’-CTGGGAAGGGTCTACCCAC-3’ | 5’-GGTGCTATGTTAGCGGCCTC-3’ |
| Ace2 | 5’-TCCAGACTCCGATCATCAAGC-3’ | 5’-GCTCATGGTGTTCAGAATTGTGT-3’ |
| Tmprss2 | 5’-CAGTCTGAGCACATCTGTCCT-3’ | 5’-CTCGGAGCATACTGAGGCA-3’ |
| Gapdh | 5’-AGGTCGGTGTGAACGGATTTG-3’ | 5’-TGTAGACCATGTAGTTGAGGTCA-3’ |

**Supplementary Figure 1.** Unprocessed scans of dot blots by Proteome Profiler Mouse XL Cytokine Array. The dot spots represent the change in cytokines in mice serum in the **a)** control (Ctrl) group, **b)** white button mushroom (WBM)-treated group, and **c)** Lentinan (LT)-treated group.


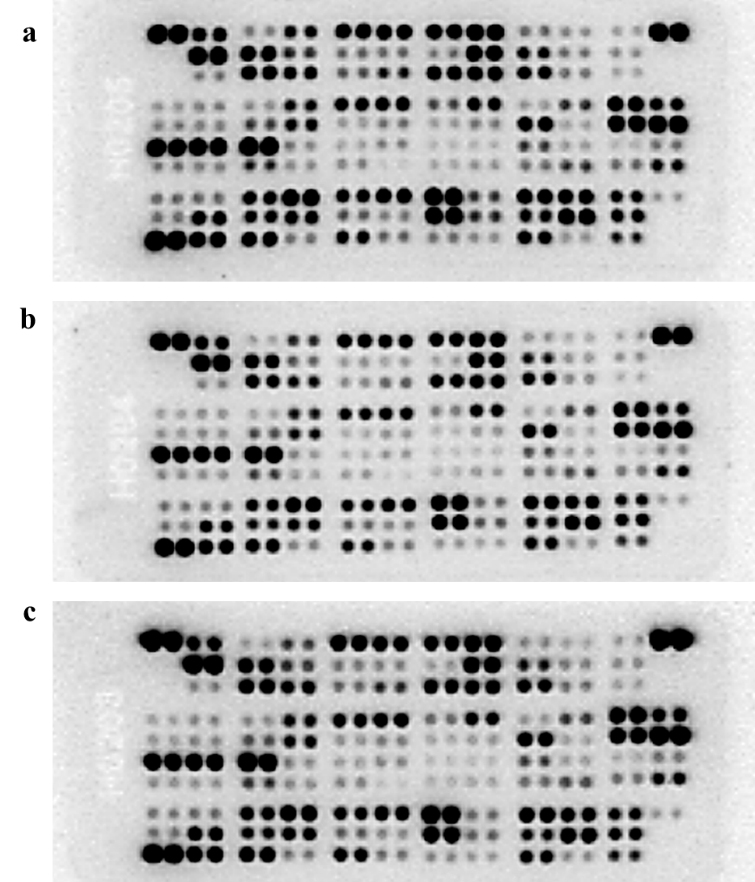


**Supplementary Figure 2.** Gating strategies used for MDSC cell sorting. We used four sorting controls to gate MDSCs in blood and spleen. These sorting controls included a non-staining blank control, a CD45-FITC staining control, a CD11b-PerCP-Cy5.5 staining control, and a Gr-1 Ly6G/Ly-6C-PE staining control. **a)** The non-staining blank control was used to select monocytes (singlets) from blood or splenocytes. **b)** The following staining controls were then used to sort the monocytes by their respective surface markers: CD45-FITC for CD45^+^ cells, CD11b-PerCP-Cy5.5 for CD11b^+^ cells, and Gr-1 Ly6G/Ly-6C-PE for Gr-1^+^ cells. **c)** CD45^+^ monocytes were further gated by CD11b-PerCP-Cy5.5 and Gr-1-Ly6G/Ly-6C-PE as monocytic MDSCs (M-MDSCs/CD45^+/^CD11b^+/^Gr-1low/mid) and granulocytic MDSCs (PMN-MDSCs/CD45^+/^CD11b^+/^Gr-1high).


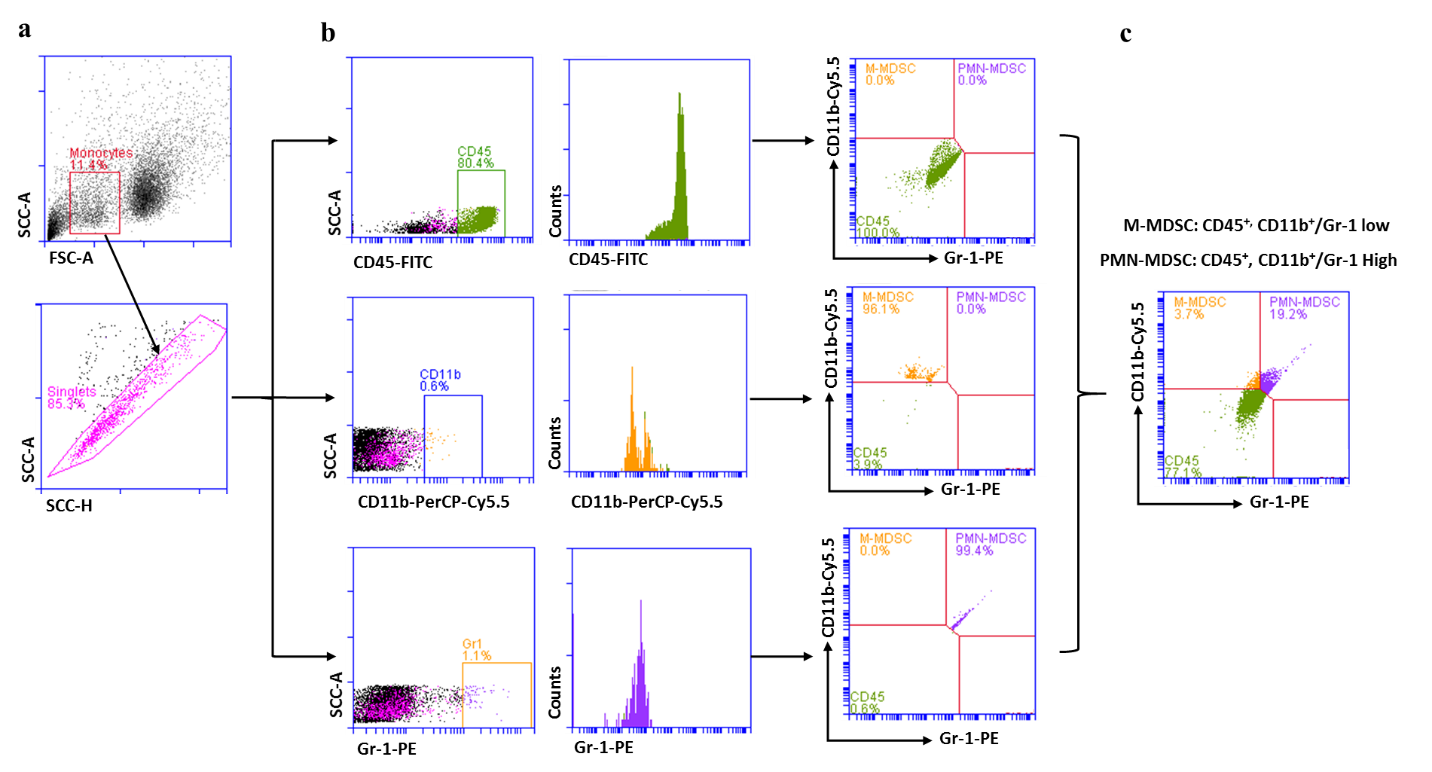

Supplement: Supplementary file 1 — Supplementary Information [file 41538_2021_102_MOESM1_ESM.docx]
