# Supplementary material for: White button mushroom interrupts tissue AR-mediated TMPRSS2 expression and attenuates pro-inflammatory cytokines in C57BL/6 mice
Source: NPJ Sci Food. 2021 Aug 2;5:20. doi: 10.1038/s41538-021-00102-6 (PMC8329194; doi:10.1038/s41538-021-00102-6)
Supplement: Supplementary file 2 — Reporting Summary [file 41538_2021_102_MOESM2_ESM.pdf]

nature portfolio

Corresponding author(s): DBPR-NPJSCIFOOD-00370 Shiuan Chen

Last updated by author(s): Jun 18, 2021

Reporting Summary

Nature Portfolio wishes to improve the reproducibility of the work that we publish. This form provides structure for consistency and transparency in reporting. For further information on Nature Portfolio policies, see our Editorial Policies and the Editorial Policy Checklist.

Statistics

For all statistical analyses, confirm that the following items are present in the figure legend, table legend, main text, or Methods section.

n/a	Confirmed	
☐	☒	The exact sample size (*n*) for each experimental group/condition, given as a discrete number and unit of measurement
☐	☒	A statement on whether measurements were taken from distinct samples or whether the same sample was measured repeatedly
☐	☒	The statistical test(s) used AND whether they are one- or two-sided<br>*Only common tests should be described solely by name; describe more complex techniques in the Methods section.*
☒	☐	A description of all covariates tested
☒	☐	A description of any assumptions or corrections, such as tests of normality and adjustment for multiple comparisons
☐	☒	A full description of the statistical parameters including central tendency (e.g. means) or other basic estimates (e.g. regression coefficient) AND variation (e.g. standard deviation) or associated estimates of uncertainty (e.g. confidence intervals)
☒	☐	For null hypothesis testing, the test statistic (e.g. F, t, r) with confidence intervals, effect sizes, degrees of freedom and P value noted<br>*Give P values as exact values whenever suitable.*
☒	☐	For Bayesian analysis, information on the choice of priors and Markov chain Monte Carlo settings
☒	☐	For hierarchical and complex designs, identification of the appropriate level for tests and full reporting of outcomes
☒	☐	Estimates of effect sizes (e.g. Cohen's d, Pearson's r), indicating how they were calculated

Our web collection on statistics for biologists contains articles on many of the points above.

Software and code

Policy information about availability of computer code

Data collection	*Provide a description of all commercial, open source and custom code used to collect the data in this study, specifying the version used OR state that no software was used.*
Data analysis	*Provide a description of all commercial, open source and custom code used to analyse the data in this study, specifying the version used OR state that no software was used.*

For manuscripts utilizing custom algorithms or software that are central to the research but not yet described in published literature, software must be made available to editors and reviewers. We strongly encourage code deposition in a community repository (e.g. GitHub). See the Nature Portfolio guidelines for submitting code & software for further information.

Data

Policy information about availability of data

All manuscripts must include a data availability statement. This statement should provide the following information, where applicable:

- Accession codes, unique identifiers, or web links for publicly available datasets
- A description of any restrictions on data availability
- For clinical datasets or third party data, please ensure that the statement adheres to our policy

The data supporting the findings reported herein are available on reasonable request from the corresponding author.

Field-specific reporting

Please select the one below that is the best fit for your research. If you are not sure, read the appropriate sections before making your selection.

☒ Life sciences ☐ Behavioural & social sciences ☐ Ecological, evolutionary & environmental sciences

For a reference copy of the document with all sections, see nature.com/documents/nr-reporting-summary-flat.pdf

Life sciences study design

All studies must disclose on these points even when the disclosure is negative.

Sample size

To study WBM-mediated AR-TMPRSS2 suppression, 12 mice were randomly divided into 4 groups (n=3 each group);
To study WBM-mediated immunomodulation, 15 mice were randomly divided into 3 groups (n=5 each group);
For the expression of AR, TMPRSS2, and ACE2 in the human prostate, lungs, small intestine, and kidneys, the mRNA expression values were obtained from "The Human Protein Atlas/HPA" (http://www.proteinatlas.org/). There were 152 samples for prostate, 427 samples for lungs, 137 samples for the small intestine, and 45 samples for kidneys.

Data exclusions

No data was excluded for analysis.

Replication

Measurements were taken from distinct samples in each treatments.

Randomization

To study WBM-mediated AR-TMPRSS2 suppression, 12 mice were randomly divided into 4 groups (n=3 each group);
To study WBM-mediated immunomodulation, 15 mice were randomly divided into 3 groups (n=5 each group);

Blinding

The studies were blinded to investigators by labeling experimental animals with randomized numbers. The sampling and analyzing were performed by two independent researches.

Reporting for specific materials, systems and methods

We require information from authors about some types of materials, experimental systems and methods used in many studies. Here, indicate whether each material, system or method listed is relevant to your study. If you are not sure if a list item applies to your research, read the appropriate section before selecting a response.

Materials & experimental systems

n/a	Involved in the study	
☐	☒	Antibodies
☒	☐	Eukaryotic cell lines
☒	☐	Palaeontology and archaeology
☐	☒	Animals and other organisms
☒	☐	Human research participants
☒	☐	Clinical data
☒	☐	Dual use research of concern

Methods

n/a	Involved in the study	
☐	☐	ChIP-seq
☐	☒	Flow cytometry
☐	☐	MRI-based neuroimaging

Antibodies

Antibodies used

AR (SP107, Sigma-Aldrich), ACE2 (ab108252, Abcam), and TMPRSS2 (ab92323, Abcam)
FITC conjugated anti-mouse CD45 (30-F11, eBioscience, San Diego, CA),
PerCP-Cy5.5 conjugated anti-mouse CD11b (M1/70, eBioscience, San Diego, CA),
PE-conjugated anti-mouse Gr-1 Ly6G/Ly-6C (RB6-8C5, eBioscience, San Diego, CA).

Validation

https://www.sigmaaldrich.com/US/en/product/sigma/200r1
https://www.abcam.com/ACE2-antibody-EPR44352-ab108252.html
https://www.abcam.com/TMPRSS2-antibody-EPR3861-ab92323.html
https://www.thermofisher.com/antibody/product/CD45-Antibody-clone-30-F11-Monoclonal/11-0451-82
https://www.thermofisher.com/antibody/product/CD11b-Antibody-clone-M1-70-Monoclonal/45-0112-82
https://www.thermofisher.com/antibody/product/Ly-6G-Ly-6C-Antibody-clone-RB6-8C5-Monoclonal/12-5931-8

Animals and other organisms

Policy information about studies involving animals; ARRIVE guidelines recommended for reporting animal research

Laboratory animals

Eight-week-old male C57BL/6 mice (The Jackson Laboratory, Sacramento, CA)

Wild animals | N/A

Field-collected samples | Mice were housed in an AAALAC (Association for Assessment and Accreditation of Laboratory Animal Care International)-accredited Animal Resources Center. Throughout the treatments, body weights were monitored every two days as an indicator of the mice's overall health. At the end of the treatments, the mice were euthanized. The prostate, lungs, small intestine, and kidneys were flash-frozen in liquid nitrogen and/or fixed with 4% paraformaldehyde. Whole blood samples were collected and isolated into serum and cell pellets. Serum samples were stored at −80°C for the cytokine array assay, while cell pellets were pre-fixed and stored at −80°C for the flow cytometry assay. Spleens were collected to isolate splenocytes, which were then pre-fixed and stored at −80°C for the flow cytometry assay.

Ethics oversight | Animal research procedures were approved by the Institutional Animal Care and Use Committee (IACUC) at City of Hope (Approval Number:, 15051)

Note that full information on the approval of the study protocol must also be provided in the manuscript.

ChIP-seq

Data deposition

☐ Confirm that both raw and final processed data have been deposited in a public database such as GEO.

☐ Confirm that you have deposited or provided access to graph files (e.g. BED files) for the called peaks.

Data access links
May remain private before publication. | *For "Initial submission" or "Revised version" documents, provide reviewer access links. For your "Final submission" document, provide a link to the deposited data.*

Files in database submission | *Provide a list of all files available in the database submission.*

Genome browser session
(e.g. UCSC) | *Provide a link to an anonymized genome browser session for "Initial submission" and "Revised version" documents only, to enable peer review. Write "no longer applicable" for "Final submission" documents.*

Methodology

Replicates | *Describe the experimental replicates, specifying number, type and replicate agreement.*

Sequencing depth | *Describe the sequencing depth for each experiment, providing the total number of reads, uniquely mapped reads, length of reads and whether they were paired- or single-end.*

Antibodies | *Describe the antibodies used for the ChIP-seq experiments; as applicable, provide supplier name, catalog number, clone name, and lot number.*

Peak calling parameters | *Specify the command line program and parameters used for read mapping and peak calling, including the ChIP, control and index files used.*

Data quality | *Describe the methods used to ensure data quality in full detail, including how many peaks are at FDR 5% and above 5-fold enrichment.*

Software | *Describe the software used to collect and analyze the ChIP-seq data. For custom code that has been deposited into a community repository, provide accession details.*

Flow Cytometry

Plots

Confirm that:

☒ The axis labels state the marker and fluorochrome used (e.g. CD4-FITC).

☒ The axis scales are clearly visible. Include numbers along axes only for bottom left plot of group (a 'group' is an analysis of identical markers).

☒ All plots are contour plots with outliers or pseudocolor plots.

☒ A numerical value for number of cells or percentage (with statistics) is provided.

Methodology

Sample preparation | Whole blood samples were collected and isolated into serum and cell pellets. Serum samples were stored at −80°C for the cytokine array assay, while cell pellets were pre-fixed and stored at −80°C for the flow cytometry assay. Spleens were collected to isolate splenocytes, which were then pre-fixed and stored at −80°C for the flow cytometry assay.

Instrument | BD Accuri C6 Plus flow cytometer (BD, San Jose, CA)

Software | BD Accuri C6 system software (BS CSampler Plus)

Cell population abundance

CD45+ monocytes account for ~80% of total monocytes (singlets);
CD11b+ monocytes account for ~0.6% of total monocytes (singlets);
Gr-1+ monocytes account for ~1% of total monocytes (singlets).

Gating strategy

We used four sorting controls to gate MDSCs in blood and spleen. These sorting controls included a non-staining blank control, a CD45-FITC staining control, a CD11b-PerCP-Cy5.5 staining control, and a Gr-1 Ly6G/Ly-6C-PE staining control. a) The non-staining blank control was used to select monocytes (singlets) from blood or splenocytes. b) The following staining controls were then used to sort the monocytes by their respective surface markers: CD45-FITC for CD45+ cells, CD11b-PerCP-Cy5.5 for CD11b+ cells, and Gr-1 Ly6G/Ly-6C-PE for Gr-1+ cells. c) CD45+ monocytes were further gated by CD11b-PerCP-Cy5.5 and Gr-1-Ly6G/Ly-6C-PE as monocytic MDSCs (M-MDSCs/CD45+/CD11b+/Gr-1low/mid) and granulocytic MDSCs (PMN-MDSCs/CD45+/CD11b+/Gr-1high).

☒ Tick this box to confirm that a figure exemplifying the gating strategy is provided in the Supplementary Information.

Magnetic resonance imaging

Experimental design

Design type

Indicate task or resting state; event-related or block design.

Design specifications

Specify the number of blocks, trials or experimental units per session and/or subject, and specify the length of each trial or block (if trials are blocked) and interval between trials.

Behavioral performance measures

State number and/or type of variables recorded (e.g. correct button press, response time) and what statistics were used to establish that the subjects were performing the task as expected (e.g. mean, range, and/or standard deviation across subjects).

Acquisition

Imaging type(s)

Specify: functional, structural, diffusion, perfusion.

Field strength

Specify in Tesla

Sequence & imaging parameters

Specify the pulse sequence type (gradient echo, spin echo, etc.), imaging type (EPI, spiral, etc.), field of view, matrix size, slice thickness, orientation and TE/TR/flip angle.

Area of acquisition

State whether a whole brain scan was used OR define the area of acquisition, describing how the region was determined.

Diffusion MRI ☐ Used ☐ Not used

Preprocessing

Preprocessing software

Provide detail on software version and revision number and on specific parameters (model/functions, brain extraction, segmentation, smoothing kernel size, etc.).

Normalization

If data were normalized/standardized, describe the approach(es): specify linear or non-linear and define image types used for transformation OR indicate that data were not normalized and explain rationale for lack of normalization.

Normalization template

Describe the template used for normalization/transformation, specifying subject space or group standardized space (e.g. original Talairach, MNI305, ICBM152) OR indicate that the data were not normalized.

Noise and artifact removal

Describe your procedure(s) for artifact and structured noise removal, specifying motion parameters, tissue signals and physiological signals (heart rate, respiration).

Volume censoring

Define your software and/or method and criteria for volume censoring, and state the extent of such censoring.

Statistical modeling & inference

Model type and settings

Specify type (mass univariate, multivariate, RSA, predictive, etc.) and describe essential details of the model at the first and second levels (e.g. fixed, random or mixed effects; drift or auto-correlation).

Effect(s) tested

Define precise effect in terms of the task or stimulus conditions instead of psychological concepts and indicate whether ANOVA or factorial designs were used.

Specify type of analysis: ☐ Whole brain ☐ ROI-based ☐ Both

Statistic type for inference
(See Eklund et al. 2016)

Specify voxel-wise or cluster-wise and report all relevant parameters for cluster-wise methods.

Correction

Describe the type of correction and how it is obtained for multiple comparisons (e.g. FWE, FDR, permutation or Monte Carlo).

Models & analysis

n/a	Involved in the study
☐	☐ Functional and/or effective connectivity
☐	☐ Graph analysis
☐	☐ Multivariate modeling or predictive analysis

Functional and/or effective connectivity	*Report the measures of dependence used and the model details (e.g. Pearson correlation, partial correlation, mutual information).*
Graph analysis	*Report the dependent variable and connectivity measure, specifying weighted graph or binarized graph, subject- or group-level, and the global and/or node summaries used (e.g. clustering coefficient, efficiency, etc.).*
Multivariate modeling and predictive analysis	*Specify independent variables, features extraction and dimension reduction, model, training and evaluation metrics.*
